# Coronal Approach for Dorsal Preservation Rhinoplasty

**DOI:** 10.1093/asjof/ojaf018.001

**Published:** 2025-05-13

**Authors:** Jake Alford, Tommy Liu, Justine Lee

**Affiliations:** University of California - Los Angeles, Los Angeles; Private Practice, Seattle, WA; UCLA, Los Angeles

## Abstract

**Goals/Purpose:**

Dorsal preservation rhinoplasty has increased in popularity over traditional structural rhinoplasty in recent year. Dorsal preservation rhinoplasty fundamentally involves maintaining the structure of the patient’s native osseocartilaginous dorsum without separating the upper lateral cartilages from the septum. Proponents of the dorsal preservation technique argue that maintaining the native dorsum during surgery allows for a more natural appearing postoperative outcome. However, reported challenges and drawbacks of the technique include lowering of the radix or creation of a step off over the dorsal hump at the level of the transverse osteotomy. The aim of the authors is to describe a modification to the dorsal preservation technique in which the osteotomy is performed under direct visualization by way of a coronal approach. The authors describe the relative advantages of this technique in their practice. Finally, the authors recommend the specific patient population and clinical scenarios in which this modification is best utilized.

**Methods/Technique:**

All patients undergoing the coronal approach to dorsal preservation rhinoplasty were patients of the senior author performed at a single institution. All patients were candidates for dorsal preservation rhinoplasty based on the senior surgeon’s preoperative assessment. All patients underwent concurrent facial feminization surgery or a coronal brow lift surgery at the time of dorsal preservation rhinoplasty. Coronal incision and dissection was performed in all patients for either facial feminization or coronal brow lift. The technique involves standard dissection in a subperiosteal plane to the nasofrontal junction. Additional dissection is then continued so that medial orbit, cranial aspect of nasal sidewall, and radix is exposed and directly visualized. 2mm osteotome is then used to create the transverse osteotomy at the level of the radix. Osteotomy is carried down onto nasal sidewall approximately one half of the length of the desired osteotomy until visualization is lost. Remaining caudal nasal osteotomy is completed through the standard rhinoplasty approach either percutaneously or internally. After the final position of the dorsum is achieved in step-down or push-down fashion, the osteotomy sites can be directly inspected for discrepancies or stepoffs.

**Results/Complications:**

Dorsal preservation rhinoplasty has the advantage of giving the patient a more naturally appearing post-operative result by maintaining the integrity of the osseocartilaginous dorsum. However, the en bloc movement and lack of individual structural dissection has the potential to lead to lack of control over the mobile segment, palpable step offs, and visible contour irregularities at the bony radix. In our experience, one solution to these challenges is use of a coronal approach. In these patients (patients undergoing concurrent procedures requiring a coronal approach), visualization while performing the transverse osteotomy prevents unwanted changes to the radix. Any contour discrepancies, bony spurs, or step offs of the cranial dorsum can be visualized and addressed as needed. An insufficiently mobile segment can be secured more easily.

**Conclusion:**

Dorsal preservation rhinoplasty is increasing in popularity and can result in a more naturally appearing outcome for the patient. While usually performed through standard rhinoplasty incisions, the authors describe a modification of this known technique that can be used when a patient is already undergoing coronal incision during a concurrent procedure. In this technique, the transverse osteotomy and majority of the lateral nasal osteotomies can be performed under direct visualization via a coronal approach. In our experience, this approach allows for optimal control over the radix, minimizes step offs and contour deformities, and better fixation of the mobile segment if needed. In a patient undergoing both rhinoplasty and a procedure requiring a coronal incision, this technique should be considered as a strategy to provide the surgeon more control over the operative result.

